# The Percy Treatment of Carcinoma of the Uterus

**Published:** 1916-09-23

**Authors:** 


### THE PERCY TREATMENT OF CARCINOMA OF THE UTERUS.

### An Ingenious Adaptation of the Cautery.

Only a few days before the outbreak of the European war a good deal of interest was aroused among British surgeons by the paper read before the American Surgical Congress in London by Dr. J. F. Percy, of Galesburg, Illinois, on the treatment of a new method of inoperable malignant disease of the uterus. But for the war this method would probably have been extensively tried in this country; and even as it is, one hears of institutions in England which are accumulating a mass of valuable data as to its results, in due time to be published with statistics, it may be hoped. Meanwhile the Percy treatment is being given a trial in America on a wide scale; and although it is too early as yet to expect any decided verdict for or against it, occasional references to it in American surgical literature are beginning to make an appearance.

### The Principles of Percy's Work.

The main principle underlying Percy's treatment is one that has been laid down by a number of research workers, including Clowes, Loeb, Haaland, Vidal, and others: namely, that carcinoma and sarcoma cells are destroyed by a rise of temperature which is variously estimated by different authors between 40° C. and 55° C., while connective-tissue cells can survive this heat, but are killed between 55° and 65° C. From this fact, if fact it be, Percy has deduced that if a moderate degree of heat can be made to suffuse the whole of a cancerous tumour, it should be possible to destroy it without at the same time causing irreparable injury to surrounding structures and subjecting the patient to serious risks of sloughing, sepsis, and hæmorrhage.

As far as we are aware, this principle of cauterisation by moderate heat ("cold cauterisation") has been applied hitherto only to malignant growths of the uterus; but obviously its application, if it turns out a success, need not be limited to neoplasms of that organ. It has, moreover, only been advocated for growths of the uterus which are too advanced for any surgical operation to have a chance of permanent success: in other words, for extensive growths too widespread even for Wertheim's operation, which has so much wider a scope than any technique previously devised for the relief of this terrible malady.

The second general principle of Percy's method is that moderate heat can be made to penetrate living tissue to a much greater distance than great heat, because it does not carbonise the surface tissues and diminish their conductivity. He claims that whereas coagulation will spread only half an inch below the surface of muscular tissue from a very hot iron, with a low grade of heat (40°-50° C.) coagulation can be procured for a distance of two and a half inches. He emphasises especially the fact that nothing is actually removed at the time of operation; the tissues are merely coagulated and slowly dissolved. His latest claim is that 90 per cent. of cases of cancer of the uterus are operable when his method is used, by which he appears to mean that a Wertheim operation is practicable in that percentage after the application of heat by his method. His criterion of the duration of the cauterisation is the mobility of the uterus; if the fixed tissues are not made movable, the heat penetration has not been sufficient, and must be continued.

### The Percy Cautery.

In order to put these two principles into practice, Percy has devised an electric cautery which is attached to a rheostat, whereby he can control the heat produced in the iron by the current flowing through it. He performs a median laparotomy, and ligatures transperitoneally the internal iliac arteries (to control subsequent hæmorrhage, if any); sometimes the uterine arteries are ligatured instead of the internal iliacs; he also ligatures the ovarian arteries. The patient is then put in the lithotomy position, and a specially invented water-cooled speculum is put into the vagina; this is to prevent possible burning of the vaginal walls during the cauterisation of the uterus. An assistant grasps the uterus through the laparotomy wound, which has been left unsutured for that purpose. The cautery iron is then introduced through the os uteri and pushed up as far as it will go into the uterus: the assistant can give the operator useful information during this process as to the position of the iron, because he can grasp the uterus in his hand. The current is then turned on, and a gradual process of "cooking" begins: that is to say, of coagulation and destruction of the cells within the hot zone radiating out from the cautery. The iron should never be allowed to get so hot that smoke is produced. At the most a gentle sizzling noise is allowed as evidence of the cauterisation. It should take about half an hour for the heat to penetrate through the uterus sufficiently to create a sensation of warmth in the (gloved) hand of the assistant. As a matter of experience it often happens that some part of the uterine wall is deeply excavated by cancerous ulceration, and then the heat of the cautery penetrates to the peritoneal surface much more quickly. The assistant should never experience an uncomfortable degree of heat: if he does, the current should be lowered. The cauterisation is continued until the uterus, which is in most cases fixed to begin with (since otherwise it would be within the scope of a Wertheim operation), becomes movable, and until all parts of it have been subjected to the hot iron. The abdominal wound is then closed; and the operation, which takes anything from one to two and a half hours, is completed.

### Some of the Results

What eventual verdict will be passed upon this method of treatment it is futile to anticipate; in the nature of things it must be three or four years before cases can have been watched sufficiently long for end results to be formulated with confidence. The fact that only inoperable and practically hopeless cases are treated naturally tries the method very high, and it is inevitable that in such cases a severe procedure such as this should be attended by a certain direct mortality. Beyond that, all that can be said is that some of those that have used the method speak encouragingly of it, and are continuing its employment. In two cases, at least, where death has speedily followed the operation, the surgeon in charge has secured a *post-mortem* examination, removed the cancerous uterus, and had it microscopically examined to decide how far Percy's technique does do what is claimed for it—that is, destroy the cancer cells. These cases were under Boldt and Bancroft respectively in America; and both have been reported.*

Boldt's findings led him into direct antagonism with Percy: he concluded that there is no evidence that low degrees of heat spread further through the cancer than high degrees, and that in his case a good many of the cancer cells had escaped injury, and were apparently still viable. (This inference is admittedly uncertain, for no one can tell positively by looking at a cancer cell under the microscope whether it was or was not viable when the specimen was taken.) Boldt's latest contribution to the subject is to the effect that Percy's low heat should be reserved principally for a second application, after rapid destruction has been accomplished with high heat and the charred eschar thus caused has been thrown off; or for those advanced cases in which the application of great heat would endanger the bladder or the rectum. Apart from this he does not admit any merit for the Percy cautery.

Bancroft is less dogmatic: he found extensive degeneration of cancer cells in his case, together with a certain but lesser amount of damage to the muscular and connective-tissue cells, and he also found at the periphery of his specimen cancer cells which were apparently unchanged. The chief points he makes are that the operation is a serious one, and may cause death: it is often followed by shock and by a high temperature subsequently. But if the immediate effects are survived (as they nearly always are) the sequelæ are not severe—less so than those of radium implantation, for instance. The connective tissue and other normal structures do not, as Percy believes, escape injury; though they are not so much affected as are the cancer cells. These inferences seem to be justified, and Bancroft's paper should be studied by any surgeon who thinks of giving the Percy treatment of inoperable cancer of the uterus a trial.

Charles Mayo, who also took part in the discussion of Bancroft's paper, seems distinctly more favourable to the "cold" cautery method, on the strength of results obtained at the Mayo Clinic. He uses guarded language, and appears to admit that his results are not absolute proof positive of Percy's contention: still, the mere fact that he has obtained good results is distinctly a feather in the latter's cap and will probably induce many more surgeons in America to give the technique a trial.

* In the *American Journal of Obstetrics*: Boldt's case in January 1916; Bancroft's in July 1916.

